# Hashimoto encephalopathy associated with hyperthyroidism: A case report

**DOI:** 10.3892/etm.2014.1761

**Published:** 2014-06-05

**Authors:** XU HUANG, YANG YU, HUA ZHANG, JIE LIU, YUQIAN SUN, MANLI CHANG, CAN CUI

**Affiliations:** Department of Endocrinology and Metabolism, The Second Hospital Affiliated to Harbin Medical University, Harbin, Heilongjiang 150080, P.R. China

**Keywords:** hashimoto encephalopathy, hashimoto thyroiditis, hyperthyroidism, corticosteroid

## Abstract

Hashimoto’s encephalopathy (HE) is an acute encephalopathy associated with Hashimoto’s thyroiditis. The majority of reported cases have been associated with hypothyroidism, while cases with hyperthyroidism are rare. The current study reported on a 56-year old female patient with HE, who was found to have progressively aggregated dysarthria, gait disturbance, somniloquy and delirium. Thyroid function tests revealed that the patient had hyperthyroidism, with high levels of anti-thyroid antibodies. Following treatment with corticosteroids, the neurological/psychiatric symptoms of the patient were relieved quickly. The one-year follow-up investigation indicated that there was no recurrence of the disease, demonstrating that the treatment administered for this rare case was effective.

## Introduction

Hashimoto’s encephalopathy (HE) is a neurological complication of autoimmune thyroid disease, which is independent of thyroid status. HE is also known as steroid-responsive encephalopathy with autoimmune thyroiditis (1,2). The condition is more frequently found in females than in males, with a ratio of ~4:1, however, the occurrence of the disease is not associated with age. Two types of initial clinical presentation may be observed for HE. Firstly, a vasculitic type with stroke-like episodes and mild cognitive impairment; and secondly, a diffuse progressive type with predominant dementia (3). Since the first report of HE by Brain *et al* in 1966 (5), the majority of HE cases have been shown to be associated with hypothyroid function. However, HE associated with hyperthyroid function is very rare (6,7). In the present study, an HE case associated with hyperthyroid function was reported.

## Case report

A 56-year-old female patient that presented with dysarthria, gait disturbance, somniloquy and delirium was admitted to the Second Hospital Affiliated to Harbin Medical University (Harbin, China) in March 2012. Prior written and informed consent was obtained from the patient and the study was approved by the Ethics Review Board of Harbin Medical University. The patient had been diagnosed with hyperthyroidism 14 years previously and had received bilateral subtotal thyroidectomy surgery 12 years previously. Following this therapy, the patient had not been administered any drugs associated with thyroid disease or presented with hyperthyroidism-associated symptoms. For three months prior to hospital admission, the patient had been suffering from diarrhea and weight loss. The condition was treated as colitis gravis and the symptom of diarrhea was remitting. At the time of admission, the body temperature of the patient was 36°C, the pulse rate was 159 beats per minute with normal regularity and the blood pressure was 140/80 mmHg. Neural-system examination revealed dysarthria and mild attenuation of the muscle force of the limbs without pathological relax.

Thyroid function tests indicated hyperthyroidism. Thyroid autoantibodies tests were positive, with particularly high titrates of anti-thyroid peroxidase antibody (TPO-Ab; Table I). In addition, ultrasonography revealed that the residual thyroid parenchyma had heterogeneous echogenicity with an abundant blood flow. Radioiodine uptake was 56% at 3 h and additional biochemical tests were all in a normal range (Table II). The disease history, symptoms and laboratory test results supported the diagnosis of hyperthyroidism. However, after two weeks of antihyperthyroidism treatment, there had been no decrease in the neurological/psychiatric symptoms.

**Figure 1:**
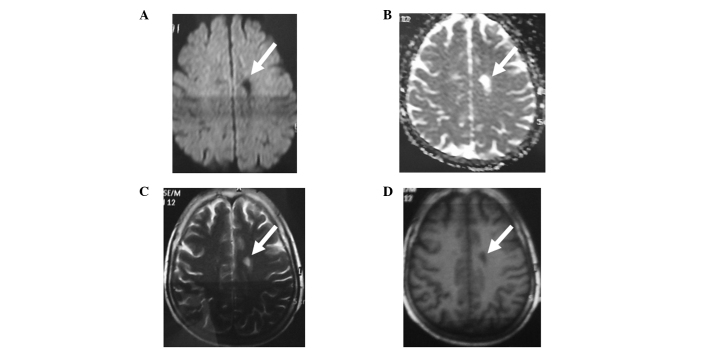
MRI images showing dissymmetry of the bilateral cerebral hemisphere. (A) DWI revealed a dark focus (white arrow) at the commissural magna cerebri. (B) ADC mapping indicated that this focus (white arrow) was of increased signal intensity. (C) T2 and (D) FLAIR images revealed areas (white arrows) of increased signal intensity corresponding to the areas of diffusion signal intensity abnormality. MRI, magnetic resonance imaging; DWI, diffusion-weighted imaging; ADC, apparent diffusion coefficient; FLAIR, fluid attenuated inversion recovery.

**Figure 2:**
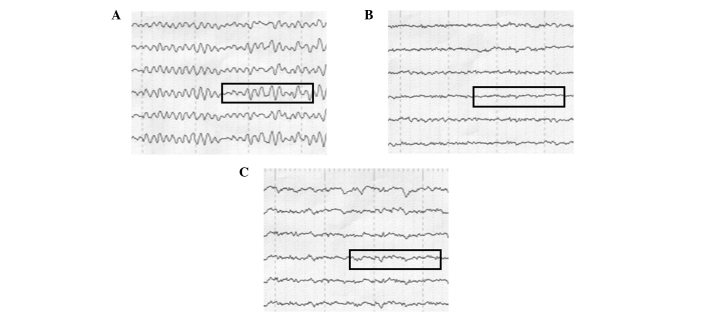
Serial EEG and BEAM scans of the patient. Representative images of the high-power θ wave at the central region of the frontal region and diffuse slow waves were observed (A) when diagnosed, (B) at the two month follow-up and (C) at the one-year follow-up examination. The boxes indicate the waves in the EEG. EEG, electroencephalograms; BEAM, brain electrical activity mapping.

Magnetic resonance imaging (MRI) of the brain revealed that there was a soft focus at the site of the commissural magna cerebri and the signals of intracranial artery vessels were rigor, uneven and inconsistent (Fig. 1). Electroencephalogram (EEG) images showed a high-power θ wave at the central region of the frontal region and diffuse slow waves (Fig. 2A). In addition, cerebrospinal fluid tests revealed that the concentration of protein was high and that there were no cells. The assay for TPO-Ab was positive in the cerebrospinal fluid (Table I), which confirmed the diagnosis of HE. After three days of methylprednisolone (500 mg/day) administration, the symptoms of somniloquy and delirium disappeared. In addition, the dysarthria and motor function improved and the patient was able to walk when aided by another person. The steroid agent was changed to oral prednisolone with a first dose of 30 mg/day (Table III) and the patient was administered corticosteroid drugs for almost three months (Table III). The neurological/psychiatric symptoms recovered and there was no recurrence during the one-year follow-up investigation. EEG examinations at the two-month and one-year follow-ups revealed no abnormal changes, with the exception of certain slow waves (Fig. 2B and C). The patient continued to receive antihyperthyroidism treatment with Methimazole (5 mg/day) while the titrates of TPO-Ab remained at a high level, despite exhibiting euthyroid function (Table I). The daily life of the patient was independent and housework was performed freely. The results indicated that the treatment administered was effective for this rare case of HE.

## Discussion

A rare case of HE associated with hyperthyroidism was reported in the present study. Although at first the patient was suspected to have thyrotoxic psychosis, increasing evidence, including results from the brain MRI, EEG, cerebrospinal fluid tests and steroid responsiveness, supported the diagnosis of a rare cases of HE that was associated with hyperthyroidism. HE is commonly characterized by neurological/psychiatric symptoms, high levels of anti-thyroid antibodies, non-specific radiological examinations or EEG abnormalities and responsiveness to corticosteroid treatment. The case reported in the present study complied with all the aforementioned symptoms. Previously, a specific HE case was shown to be clinically and biochemically euthyroid, exhibiting onset symptoms similar to presenile dementia (8).

With regard to the current case, the TPO-Ab assay was also positive in the cerebrospinal fluid, which may be more sensitive compared with serum antibody detection. Although the role of thyroid autoantibodies is unclear, HE is a type of autoimmune encephalopathy. The majority of patients respond to treatment with steroids, while certain patients undergo spontaneous remission without steroid administration (9) and other patients fail to improve with steroid treatment (10). Although HE is a rare disease, the condition typically represents autoimmune encephalopathy. In the present study, the one-year follow-up results indicated that the treatment administered for this rare HE case was effective.

## Figures and Tables

**Table I:** Thyroid function test results at the baseline and during the one-year follow-up.

Time points	FT_3_ (pmol/l) (2.63–5.7)	FT_4_ (pmol/l) (9.01–19.5)	TSH (μIU/l) (0.35–4.94)	Tg-Ab (IU/ml) (0–4.11)	Tpo-Ab (IU/ml) (0–5.61)
Mar 12, 2012	35.44	61.23	0.0010	20.56	905.58
May 27, 2012	6.93	33.99	0.012	5.67	350.38
Aug 26, 2012	5.45	14.36	0.0380	9.87	550.32
Nov 14, 2012	6.86	19.17	0.0011	40.97	>1000
Jan 12, 2013	4.59	16.67	0.23	8.32	458.9
May 2, 2013	3.78	15.67	1.34	7.78	256.45

FT_3_, free triiodothyronine; FT_4_, free thyroxine; TSH, thyroid-stimulating hormone; Tg-Ab, thyroglobulin antibody; TPO-Ab, thyroid peroxidase antibody. Numbers in parentheses indicate the normal ranges.

**Table II:** Laboratory test results of the patients.

Investigations	Results	Reference range
Hemoglobin (g/l)	129	120–150
Creatinine (μmol/l)	59	49–110
Aspartate aminotransferase (IU/l)	26	0–40
Alkaline phosphatase (U/l)	92	35–150
Serum potassium (mmol/l)	4.36	3.5–5.5
Serum calcium (mmol/l)	2.2	2.1–2.7
Serum magnesium (mmol/l)	1.03	0.7–1.25
Fasting plasma glucose (mmol/l)	4.9	3.9–6.1
ANCA	<1:10	1:10
ANA	Negative	<1:100
RF (IU/ml)	12.2	0–15
CRP (mg/ml)	4.32	0–5
Anti-HIV-1/-2	Negative	-
HbsAg	Negative	-
CEA (ng/ml)	3.1	<5
TRAB (IU/ml)	42.89	0.11–30
Cerebrospinal fluid		
Protein (mmol/l)	1056	150–450
Glucose (mmol/l)	3.6	2.8–4.4
Culture	Negative	-
Gram stain	Negative	-
TPO-Ab	Positive	-

ANCA, anti-neutrophil cytoplasmic antibody; ANA, anti-nuclear antibody; HIV, human immunodeficiency virus; HbsAg, hepatitis B surface antigen; CEA, carcinoembryonic antigen; TRAB, thyrotrophin receptor antibody; CRP, C-reactive protein; RF, rheumatoid factor; TPO-Ab, thyroid peroxidase antibody.

**Table III:** Steroid treatments for the patient.

Treatments (mg/days)	Duration (days)
Intravenous pulse methylprednisolone	
500	3
Dose of oral prednisolone	
30	10
25	10
20	10
15	10
10	10
5	30
